# Study of sexual assault cases among below 18 years age group during September 2018 to September 2020 in Government Medical College, Patiala, Punjab, India: cross-sectional study

**DOI:** 10.11604/pamj.2022.41.15.29852

**Published:** 2022-01-06

**Authors:** Akash Deep Aggarwal, Preetinder Singh, Didar Singh Walia, Sumit Kukreja

**Affiliations:** 1 Department of Forensic Medicine and Toxicology, Government Medical College, Patiala, Punjab, India

**Keywords:** Child abuse, examination, fear, rape, sexual assault, trauma

## Abstract

**Introduction:**

there are instances commonly reported where sexual offences are committed against persons below 18 years of age, who are deemed as children. The adverse effects of child sexual abuse are psychological, physical, behavioural and interpersonal.

**Methods:**

this study was conducted in the casualty unit over a period of two years (September 2018 to September 2020), total 35 cases of sexual assault examined out of these 21 (60%) cases fall under the category where below 18 years age group is victim.

**Results:**

out of 21 cases, 19 (90.47%) were of female sexual assault and 2 (9.53%) were of male sexual assault. The age of victim ranged from 2 years to 18 years. Median age of the study population is 14 years. Interquartile range is 6 years. The most vulnerable age group was 12-18 years (71.4%). Most of the victims were Hindus (47.6%) and Sikhs (47.6%). 61.9 % cases belong to rural background. In 71.5% of cases, the act was committed by familiar persons. Maximum 28.5% cases were medico-legally examined within one day of incidence. Hymen was torn in only 5 (26.3%) cases.

**Conclusion:**

young girls are found out to be most vulnerable group. Young girls should receive special attention especially from poor settlements and lower social strata. Most important is that the general attitude of society needs to be changed in favors of the dignity of women and children. Social awareness, sensitization and protection programs should be a high priority.

## Introduction

Child sexual abuse has been publicly acknowledged as a problem in India and has led to the enactment of special law, Protection of Children against Sexual Offences (POCSO) 2012, which has criminalized a range of acts including child rape, harassment, and exploitation for pornography [1]. It deals with sexual offences against persons below 18 years of age, who are deemed as children. The act for the first time, defines penetrative sexual assault, sexual assault and sexual harassment. This act is a comprehensive law to protect children from such offences, while safeguarding the interests of the child at every stage of the judicial process by incorporating child-friendly mechanisms for reporting, recording of evidence, investigation and speedy trial of offences through the appointment of special public prosecutors and designated special courts [2]. The World Health Organization in 2002 estimated that 73 million boys and 150 million girls under the age of 18 years had experienced various forms of sexual violence [3]. For every 155^th^ minute a child, less than 16 years is raped, for every 13^th^ hour a child under 10, and one in every 10 children are sexually abused at any point of time [4]. Sexual violence is usually under-reported because of fear: fear of physical examination, disclosure of sexual history, repeating the traumatic experience in full detail over and over again, complicated legal procedures, not being believed by others, and being harmed by the accused [3]. The adverse effects of child sexual abuse are psychological, physical, behavioral and interpersonal [5]. Another factor which contributes is that there is a general distrust towards medical and law enforcement personnel who play vital roles in the aftermath of a sexual assault [6]. The objective of our study is to analyze and compare the magnitude of child sexual assault cases in our region and its analysis in reference to sex, age groups, religion, region, relationship with accused, delay in reporting and results of examination. The main obstacle we have in mind is the social barrier which leads to under reporting or delayed reporting of cases.

## Methods

This cross-sectional study was conducted in the casualty unit of Govt. Medical College, Patiala, Punjab, India over a period of two years (September 2018 to September 2020), and twenty one cases reported. Our institution caters to the urban region of the Patiala city along with its adjoining rural areas of the Patiala District in the State of Punjab. All cases of alleged sexual assault, whether reporting on their own or through police, were examined medico-legally and further examined radiologically to ascertain the age of victim so that bias due to wrong information about age can be ruled out and also gynecologically. Only the cases found to be of below 18 years are included in this study. The details pertaining to factors such as sex, age, religion, profession, relationship with accused, time interval between alleged incidence and medical examinations, local findings and results of examination of exhibits were recorded and analyzed statistically by grouping in table form on the basis of various factors. If at any stage the patient refuses to give history or refuses to give consent for medico legal examination that case will be excluded from study.

## Results

A total of 2358 medico-legal cases were examined in our department over the period of two years and a total 35 cases of sexual assault examined out of these 21 (60%) cases fall under POCSO Act. The most vulnerable age group was 12-18 years (71.4%) followed by 6-12 years (23.8%). Out of 21 cases, 19 (90.47%) were of females and 2 (9.53%) were of males (Table 1). The ages of victims ranged from 2 years to 18 years. Median age of the study population is 14 years. Interquartile range is 6 years. Out of these, 13 (61.9%) cases belong to rural background and 8 cases (38.1%) cases belonged to urban areas. Most of the victims were Hindus (10, 47.6%) and Sikhs (10, 47.6%) and only 1 case (4.8%) belonged to Muslim religion. As our study is based on children, so all victims were school going children or school drop outs. In one case victim was 2 year baby girl. Only 2 victims came positive for spermatozoa one male and other female, which constitute 5.8% of total victims. In 15 (71.5%) of cases, the act was committed by familiar persons, while only 6 (28.5%) cases assailants were unfamiliar to the victims. In 9 (42.9%) cases, the alleged accused were neighbours followed by friends in 3 (14.3%), relatives in 2 (9.5%). With reference to delay in reporting of incidence, only 6 (28.5%) cases were medico-legally examined within one day of incidence, followed by variable distribution and one case (4.8%) reported about one month after incidence. Among cases of female, hymen was torn in only 5 (26.3%) cases whereas one patient refused examination (Table 2). In 4 cases which are excluded from study victims refused to give consent for medico-legal examination.

**Table 1:** age and sex distribution of the victims

Age (in years)	Male		Female		Total	
	N	%	N	%	N	%
0-6	1	4.8	0	0	1	4.8
6-12	1	4.8	4	19	5	23.8
12-18	0	0	15	71.4	15	71.4
Total	2	9.6	19	90.4	21	100

**Table 2:** various contributing factors relating to the assault

Factors		Victims	
		N	%
Relation of victim with the assailant	Neighbour	9	42.9%
	Friend	3	14.3%
	Relative	2	9.5%
	Father	1	4.8%
	Stranger	6	28.5%
	**Total**	**21**	**100%**
Time interval between the alleged incidence and the medical examination	Within one day	6	28.5
	About two days	3	14.3
	About three days	2	9.5
	About four days	2	9.5
	About a week	2	9.5
	About 15 days	2	9.5
	About a month	1	4.8
	Can't say	3	14.3
	**Total**	**21**	**100%**
Condition of hymen	Intact	13	68.4
	Torn	5	26.3
	Refused examination	1	5.3
	**Total**	**19**	**100**

## Discussion

The objective of our study is to analyze and compare the magnitude of child sexual assault cases in our region and its analysis in reference to various variables. Young age group females found to be most vulnerable. Most victims belong to rural background and are of Hindu and Sikh religion mostly. In most number of cases act was committed by familiar persons. Delay in reporting also seen in many cases. Young age groups are more vulnerable also seen in other studies done in South Africa [7], Denmark [8] etc. (Table 3). As per population distribution most number of cases belong to Hindu and Sikh religion. Comparing it with other studies like Himachal Pradesh [9] 85.71% of victims were Hindus. Out of 21 cases, 19 (90.4%) are females and 2 (9.6%) are males while in various studies done in Michigan USA [10], Massachusetts USA [11], Padua Italy [12] and Lahore [13] reportedly all victims were females. Only 5.8% came positive for spermatozoa in contrast positive semen analysis was reported in 55% cases in Padua, Italy [12], 98.35% in Lahore, Pakistan [13] and only 20% in Arkansas, America [14]. Methods of sampling should be improved. Furthermore, systematic training courses for health-care professionals should also cover the need to obtain and analyze forensic evidence as well as medical reports, which together account for a substantial part of the evidence presented in court. Improving the methods for collecting patients´ stories and conducting medical examination, as well as a sensitization of healthcare staff towards patients´ physical and emotional needs would enable more detailed in-formation to be obtained from these patients, even when they are uncooperative [12]. In majority of cases, assailants are known to victim. Similar findings are seen in studies conducted in Norway [15] where 69% victims knew their offender, in Pakistan [13] 57% cases were known to the victim, in Nigeria [16] assailant were known in 57% cases, in Maharashtra [17] 83.28% assailants were known, in Mumbai [18] 93.42% were known to the victim and in Himachal Pradesh [9] 80% of the victims knew the assailant (Table 3).

**Table 3:** comparison with other study in reference to age and assailant being known

Study location	Author	Year	Commonest age	Assailant known
Patiala, Punjab	Current	2020	60% (0-18yrs)	71.5%
Western Maharashtra	Bhoi *et al*.	2017	59.6% (11-20yrs)	83.38%
Northern Himachal Pradesh	Pal *et al*.	2015	42.85% (11-20yrs)	80%
South Africa	Jewkes *et al*.	2009	43% (13-17 yrs)	--
Lahore, Pakistan	Hassan *et al*.	2007	43% (13-17yrs)	82%
Skoto, Nigeria	Greenfeld *et al*.	1997	61.8% (0-12)	57%
Copenhagen, Denmark	Larsen *et al*.	2015	66.66 (15-24yrs)	75%
Mumbai, Maharashtra	Haridas *et al*.	2016	41.44% (16-20)	93.4%
Trondheim, Norway	Haugen *et al*.	2005	-	69%

In majority of cases there is delay in reporting of incidence similarly a study from Pakistan [13] observed that 76% cases presented for medical examination after a delay of more than 72 hours. In comparison in developed countries like USA [11] median time of presentation was 16 hours. Delay in reporting of cases may be due to indecisiveness on the part of the victim, victim's parents and relatives to report cases fear of offender and indignity, fear of parents/ guardian social stigma, the gap in communication between parents and children about the issue and some circumstances victim eloped with accused to another place [19]. In our study hymen was torn in only 5 (26.3%) cases. In comparison studies from South Africa [10] showed that anogenital and hymen injuries are seen in 83% of adolescents, in Mumbai [18] 91.44% cases the hymen showed old tears at multiple positions, in Himachal Pradesh [9] 88.57% cases had hymen ruptured. There is delay in reporting of the cases or even no reporting in some cases due to social stigma leading to negative tests. Delay also leads to disappearance of various forensic evidences. There is a general distrust towards medical and law enforcement personnel who play vital roles in the aftermath of a sexual assault. Sexual violence is usually under-reported.

## Conclusion

Young girls should receive special attention especially from poor settlements and lower social strata. Most important is that the general attitude of society needs to be changed in favors of the dignity of women and children. Hence, spreading of awareness to encourage early reporting, stringent punishment to the perpetrators and immediate proper care and protection of such innocent victims remain key factors to deal with this heinous crime. In order to understand the dynamics of the episode in rape cases and to avoid discrepancies between medical reports and legal reconstructions of sexual crimes, it is crucial to provide victims with support, and to maximize their confidence in the healthcare providers who first attend to them, usually at the emergency.

### What is known about this topic

- The adolescent age group is most vulnerable to sexual violence;
- Sexual violence is usually under-reported because of fear: fear of physical examination, disclosure of sexual history, repeating the traumatic experience in full detail over and over again, complicated legal procedures, not being believed by others, and being harmed by the accused.

### What this study adds

- Methods of sampling should be improved as spermatozoa test positive only in 2 percent cases;
- There is delay in reporting at hospitals. Victims should be sensitized for early reporting in majority of cases, assailants are known to victim;
- Sexual education should be done during schooling as here are many adolescent victims.
